# Association study of relationships of polymorphisms in the *miR-21*, *miR-26b, miR-221/222* and *miR-126* genes with cervical intraepithelial neoplasia and cervical cancer

**DOI:** 10.1186/s12885-021-08743-2

**Published:** 2021-09-07

**Authors:** Jia Yang, Zhiling Yan, Yingying Wang, Jinmei Xu, Rui Li, Chuanyin Li, Shuyuan Liu, Li Shi, Yufeng Yao

**Affiliations:** 1 Institute of Medical Biology, Chinese Academy of Medical Sciences & Peking Union Medical College, Kunming, 650118 Yunnan China; 2 Department of Gynaecologic Oncology, The 3rd Affiliated Hospital of Kunming Medical University, Kunming, 650118 China; 3 Department of Obstetrics and Gynaecologic, Kunming Yan’an Hospital, Kunming, 650051 China; 4 Yunnan Key Laboratory of Vaccine Research & Development on Severe Infectious Disease, Kunming, 650118 Yunnan China

**Keywords:** Cervical intraepithelial neoplasia (CIN), Cervical cancer (CC), MiRNA, Single-nucleotide polymorphisms (SNPs), Chinese population

## Abstract

**Background:**

*miR-21*, *miR-26b*, *miR-221/222* and *miR-126* play crucial roles in cervical cancer development. Studies have shown that polymorphisms in miRNA genes can affect miRNA expression, which might be associated with cancer development.

**Methods:**

Ten single-nucleotide polymorphisms (SNPs) in the *miR-21*, *miR-26b*, *miR-221/222* and *miR-126* genes (rs1292037, rs13137 in *miR-21*; rs2227255, rs2227258 in *miR-26b*; rs2858061, rs34678647, rs2858060, rs2745709 in *miR-221/222*; rs2297537, rs2297538 in *miR-126*) were selected, and genotyped in a total of 2176 individuals, including 435 patients with cervical intraepithelial neoplasia (CIN), 743 patients with cervical cancer (CC) and 998 healthy persons using TaqMan assays, and their associations with CIN and CC were evaluated.

**Results:**

Our results showed significant differences for the rs2297538 genotypes between the CIN and CC groups (*P* = 0.001). In addition, our results also showed significant differences for the rs2297537 alleles between the CIN and CC groups (*P* = 0.003), and the C allele of rs2297537 might be associated with a decreased risk of CC (*OR* = 0.72, *95%CI*: 0.58–0.90). At the inheritance analysis, between the CIN and control groups, the T/T-T/C genotype in rs1292037 and A/A-A/T genotype in rs13137 might be associated with an increased risk of CIN in the recessive model (OR = 1.61, 95% CI: 1.17–2.20 and OR = 1.58, 95% CI: 1.15–2.15). In addition, the C/C-T/T genotype of rs2745709 might be associated with a decreased risk of CIN in the overdominant model (OR = 0.66, 95% CI: 0.52–0.82). Between, CIN and CC group, the T/T-C/C genotype in rs1292037 and A/A-T/T genotype in rs13137 might be associated with an increased risk of CC in the overdominant model (OR = 1.43, 95% CI: 1.12–1.81 and OR = 1.42, 95% CI: 1.12–1.80). The rs2297538 G/G-A/G genotype might be associated with an increased risk of CC in the recessive model (OR = 2.83, 95% CI: 1.52–5.25). The rs2297537 2C/C + C/G genotype might be associated with a decreased risk of CC (OR = 0.71, 95% CI: 0.57–0.89) in the log-additive model. The rs2745709 T/T-C/C genotype might be associated with an increased risk of CC (OR = 1.44, 95% CI: 1.13–1.83) in the overdominant model.

**Conclusion:**

Our results indicate that rs2297538 and rs2297537 in *miR-126*, rs1292037 and rs13137 in *miR-21*, and rs2745709 in *miR-221/222*, may have important roles in the development of CIN or CC.

## Backgroud

Cervical cancer (CC) is the leading cause of death from cancer in women worldwide, especially in developing countries [1]. Persistent infection with high-risk human papillomavirus (HR-HPV) is necessary for the development of CC [2]. The progression of cervical cancer can be divided into two main steps, cervical intraepithelial neoplasia (CIN) and CC [3]. In addition to HR-HPV, host genetic factors, such as miRNAs, play important roles in the development of CIN and CC [4].

MiRNAs are a class of small, noncoding single-stranded RNA molecules with approximately 20–24 nucleotides in length [5]. MiRNAs function as the regulators in cell biological process [6], especially in the development of human cancers [7, 8], including CC [9, 10]. Several studies revealed that *miR-21*, *miR-26b*, *miR-221/222* and *miR-126* are dysregulated in CC tissues and function as either tumour suppressors or tumour promoters in CC [11–16]. For example, miR-126, which is a tumour suppressor in CC, inhibits cell proliferation, migration and invasion by regulating various target genes and signalling pathways [17, 18].

Single-nucleotide polymorphisms (SNPs) are the most common type of human heritable variation [19]. SNPs in miRNA genes may affect the mature miRNA level or the binding of miRNAs to their target genes, and finally be related to the development of cancers [20–22]. In 2019, our previous study found that rs4636297 in *miR-126* is associated with CIN and CC in a Han Chinese population; the findings indicated that the T allele confers a higher risk of developing CIN and CC [23]. The association of the SNP rs4636297 with CC might due to that this SNP is related to Drosha’s recognition and cleavage of pri-miRNA [24]. In addition, Zhang et al. in 2018 reported that SNP rs1292037 is associated with the chemoresistance to cisplatin plus paclitaxel and prognosis of patients with CC [25].. Therefore, SNPs in miRNAs may alter the expression of miRNAs or affect their interaction with target genes, and ultimately be associated with cancer susceptibility.

In the current study, we genotyped 10 SNPs (rs1292037, rs13137 in *miR-21*; rs2227255, rs2227258 in *miR-26b*; rs2858061, rs34678647, rs2858060, rs2745709 in *miR-221/222*; rs2297537, rs2297538 in *miR-126*) in healthy control, CIN and CC groups to investigate associations with CIN and CC in a Han Chinese population.

## Material and methods

### Ethics statement

The current study obtained the approval of the Institutional Review Board of the No. 3 Affiliated Hospital of Kunming Medical University. The protocol used by this investigation was in accordance with the principles expressed in the Helsinki Declaration of 1975, which was revised in 2008. Written informed consent was obtained from each participant.

### Subjects and clinical information

In total, 435 patients with CIN and 743 with CC were enrolled in the current study. The patients were diagnosed with CIN and CC according to “Diagnosis and Treatment: Obstetrics and Gynaecology” and International Federation of Gynaecology and Obstetrics (FIGO 2009) at the Third Affiliated Hospital of Kunming Medical University from July 2018 to May 2020. Patients with other malignancies, a tumour therapy history and other chronic diseases were excluded. During the same period, 998 healthy women were recruited among those seeking health checkups in the same hospital and enrolled as the healthy control group.

### SNP selection and genotyping

Previous studies have demonstrated that *miR-21*, *miR-26b*, *miR-221/222* and *miR-126* are associated with CC development [11–16]. We therefore performed a preliminary survival analysis via a RNA interactomes database (ENCORI, http://starbase.sysu.edu.cn/index.php) [26], and found the expressions of these miRNA were associated with the survival rate of cervical cancer. Thus, in the current study, 10 SNPs (rs1292037, rs13137 in *miR-21*; rs2227255, rs2227258 in *miR-26b*; rs2858061, rs34678647, rs2858060, rs2745709 in *miR-221/222*; rs2297537, rs2297538 in *miR-126*) located 2 kb up or downstream of *miR-21*, *miR-26b*, *miR-221/222*, and *miR-126* were selected and the association of these SNPs with susceptibility of CIN and CC was analysed.

Genomic DNA was obtained from EDTA anti-coagulated whole blood of the subjects using QIAamp Blood Mini Kit (Qiagen NV, Venlo, the Netherlands). The probes and primers used for genotyping were all purchased from ABI (http://www.appliedbiosystems.com). The 10 SNPs were genotyped using the TaqMan fluorescent quantitative PCR method with the QuantStudio™ Real-Time PCR instrument. The total PCR volume was 5 μL, and the reaction conditions were 95 °C pre-denaturation for 10 min, 40 cycles of 95 °C denaturation for 15 s, 60 °C annealing for 1 min, and finally 60 °C extension for 5 min. Deionized water was used to replace template DNA as a negative control. The data were analysed by TaqMan Genotyper Software (Version 1.3.1). To identify the accuracy of SNP genotyping using the TaqMan assay, samples with each genotype of the 10 SNPs were sequenced.

### Statistical analysis

Statistical analysis was performed using Microsoft Excel software and the SPSS 19.0 statistical package. The Hardy-Weinberg equilibrium (HWE) of the control group was evaluated to assess the representativeness of the study population, and significance threshold was set at *P* < 0.05. Differences in age among the CIN, CC and control groups were compared using one-way ANOVA with the LSD test for multiple comparison correction. Different distributions of the SNP and miRNA alleles in the CIN, CC and control groups were compared using the chi-square test, and odds ratios (ORs) with associated 95% confidence intervals (CIs) were calculated. The association of the genotypes of these SNPs with CIN and CC was examined using inheritance model analysis in SNPstats software [27]. Five inheritance models (codominant, dominant, recessive, overdominant and log-additive) were analysed, and the best fit inheritance model of each SNP was determined based on AIC and BIC values. The inheritance model with the lowest AIC and BIC value was considered the best fit inheritance model. Bonferroni correction was performed for multiple comparisons, and the significance threshold was set at *P* < 0.005 (0.05/10).

## Results

### Clinical characteristic of subjects

A total of 2176 subjects were enrolled in the current study. The general characteristics of the subjects are presented in Table 1. The ages of the subjects showed no significant difference among the control, CIN and CC groups (*P* = 0.172). Among the 743 patients with CC, 128 had adenocarcinoma, 609 squamous cell carcinoma, and 6 adenocarcinoma and squamous cell carcinoma.

**Table 1:** The characteristics of the subjects enrolled in the current study

	Control	CIN	Cervical Cancer	*P* value
N	998	435	743	
Ages	48.05 ± 10.69	47.11 ± 11.47	47.25 ± 9.97	0.172
CIN1		74		
CIN2		47		
CIN3		314		
Histological types				
SCC			609	
AC			128	
Others			6	
Clinical stages				
I			488	
II			228	
III			24	
IV			3	

### Association of SNPs in the *miR-21*, *miR-26b*, *miR-221/222* and *miR-126* genes with control, CIN and CC

There are nine SNPs in the *miR-21*, *miR-26b*, *miR-221/222* and *miR-126* genes were found in HWE in the control group, except for rs2297538 in miR-126 (*P* = 0.026). The allelic and genotypic distributions of these 10 SNPs among the control, CIN and CC groups are presented in Table 2.

**Table 2:** The allele and genotype distribution of the SNPs in control, CIN and cervical cancer groups

SNPs	Alleles/Genotypes	Control (*n* = 998)	CIN (*n* = 435)	CC (*n* = 743)	CIN vs Control		CC vs Control		CC vs CIN	
		n (%)	n (%)	n (%)	*P* value	OR(95%CI)	*P* value	OR(95%CI)	*P* value	OR(95%CI)
rs1292037	T	1123 (56.3)	513 (59.0)	875 (58.9)	0.179	0.90 [0.76–1.05]	0.122	0.90 [0.78–1.43]	0.969	1.00 [0.85–1.19]
	C	873 (43.7)	357 (41.0)	611 (41.1)						
	T/T	329 (33.0)	138 (31.7)	268 (36.1)	0.004		0.320		0.010	
	T/C	465 (46.6)	237 (54.5)	339 (45.6)						
	C/C	204 (20.4)	60 (13.8)	136 (18.3)						
rs13137	A	1121 (56.2)	512 (58.9)	874 (58.8)	0.181	1.12 [0.95–1.31]	0.117	1.11 [0.97–1.28]	0.987	1.00 [0.84–1.18]
	T	875 (43.8)	358 (41.1)	612 (41.2)						
	A/A	327 (32.8)	138 (31.7)	268 (36.1)	0.006		0.303		0.011	
	A/T	467 (46.8)	236 (54.3)	338 (45.5)						
	T/T	204 (20.4)	61 (14.0)	137 (18.4)						
rs2858061	G	1578 (79.1)	691 (79.4)	1149 (77.3)	0.824	0.98 [0.80–1.19]	0.219	1.11 [0.94–1.30]	0.233	1.13 [0.92–1.39]
	C	418 (20.9)	179 (20.6)	337 (22.7)						
	G/G	628 (62.9)	271 (62.3)	450 (60.6)	0.436		0.453		0.171	
	G/C	322 (32.3)	149 (34.3)	249 (33.5)						
	C/C	48 (4.8)	15 (3.4)	44 (5.9)						
rs34678647	G	1615 (80.9)	684 (78.6)	1207 (81.2)	0.157	0.87 [0.71–1.06]	0.816	1.02 [0.86–1.21]	0.125	1.18 [0.96–1.45]
	T	381 (19.1)	186 (21.4)	279 (18.8)						
	G/G	662 (66.3)	263 (60.5)	490 (65.9)	0.020		0.509		0.125	
	G/T	291 (29.2)	158 (36.3)	227 (30.6)						
	T/T	45 (4.5)	14 (3.2)	26 (3.5)						
rs2858060	C	1600 (80.2)	695 (79.9)	1162 (78.2)	0.865	0.98 [0.81–1.99]	0.157	0.89 [0.75–1.05]	0.333	0.90 [0.73–1.11]
	G	396 (19.8)	175 (20.1)	324 (21.8)						
	C/C	642 (64.3)	274 (63.0)	455 (61.2)	0.603		0.369		0.397	
	C/G	316 (31.7)	147 (33.8)	252 (33.9)						
	G/G	40 (4.0)	14 (3.2)	36 (4.8)						
rs2745709	C	1229 (61.6)	507 (58.3)	878 (59.1)	0.097	0.87 [0.74–1.03]	0.137	0.90 [0.79–1.03]	0.7	1.03 [0.87–1.22]
	T	767 (38.4)	363 (41.7)	608 (40.9)						
	C/C	381 (38.2)	129 (29.7)	260 (35.0)	0.001		0.331		0.010	
	C/T	467 (46.8)	249 (57.2)	358 (48.2)						
	T/T	150 (15.0)	57 (13.1)	125 (16.8)						
rs2297537	C	1618 (81.1)	729 (83.8)	1172 (78.9)	0.081	1.21 [0.98–1.49]	0.109	0.87 [0.74–1.03]	0.003	0.72 [0.58–0.90]
	G	378 (18.9)	141 (16.2)	314 (21.1)						
	C/C	659 (66.0)	302 (69.4)	459 (61.8)	0.098		0.174		0.010	
	C/G	300 (30.1)	125 (28.7)	254 (34.2)						
	G/G	39 (3.9)	8 (1.8)	30 (4.0)						
rs2297538	G	1705 (85.4)	723 (83.1)	1261 (84.9)	0.113	1.19 [0.96–1.48]	0.644	1.05 [0.87–1.26]	0.259	0.88 [0.70–1.10]
	A	291 (14.6)	147 (16.9)	225 (15.1)						
	G/G	737 (73.8)	315 (72.4)	535 (72.0)	0.016		0.339		0.001	
	G/A	231 (23.1)	93 (21.4)	191 (25.7)						
	A/A	30 (3.0)	27 (6.2)	17 (2.3)						
rs2227255	T	1376 (68.9)	600 (69.0)	1005 (67.6)	0.988	1.00 [0.84–1.19]	0.412	1.06 [0.92–1.23]	0.502	1.06 [0.89–1.28]
	C	620 (31.1)	270 (31.0)	481 (32.4)						
	T/T	474 (47.5)	205 (47.1)	350 (47.1)	0.946		0.309		0.329	
	T/C	428 (42.9)	190 (43.7)	305 (41.0)						
	C/C	96 (9.6)	40 (9.2)	88 (11.8)						
rs2227258	G	1387 (69.5)	604 (69.4)	1002 (67.4)	0.973	1.00 [0.84–1.19]	0.195	1.10 [0.95–1.27]	0.316	1.10 [0.92–1.31]
	A	609 (30.5)	266 (30.6)	484 (32.6)						
	G/G	481 (48.2)	207 (47.6)	349 (47.0)	0.911		0.147		0.183	
	G/A	425 (42.6)	190 (43.7)	304 (40.9)						
	A/A	92 (9.2)	38 (8.7)	90 (12.1)						

The genotype distributions of rs1292037 and rs13137 in *miR-21*, rs2745709 in *miR-221/222* and rs2297537 and rs2297538 in *miR-126* differed between the CIN and CC groups (*P* < 0.05). However, only rs2297538 in *miR-126* showed a difference after Bonferroni correction (*P* = 0.001). In addition, the allele distribution of rs2297537 in *miR-126* was significantly different (*P* = 0.003), and the C allele might be associated with a decreased risk of CC (OR = 0.72, 95% CI: 0.58–0.90). In addition, the genotype distributions of rs1292037 and rs13137 in *miR-21* and rs2745709 in *miR-221/222* showed differences between the CIN and control groups (*P* < 0.05). Nevertheless, only rs1292037 in *miR-21* and rs2745709 in *miR-221/222* remained significantly differences after Bonferroni correction (*P* = 0.004 and *P* = 0.001). No difference in the distribution of these SNPs was found by comparing the control and CC groups (*P* > 0.005).

### Inheritance analysis of SNPs in the *miR-21*, *miR-26b*, *miR-221/222* and *miR-126* genes with control, CIN and CC

The association of the genotypes of the 10 SNPs with control and CIN was evaluated using inheritance model analysis (Table 3). In *miR-21*, a significant difference in rs1292037 and rs13137 was observed in the recessive model (*P* = 0.002 and 0.003). Moreover, T/T-T/C genotype in rs1292037 and A/A-A/T genotype in rs13137 might be associated with an increased risk of CIN (OR = 1.61, 95% CI: 1.17–2.20 and OR = 1.58, 95% CI: 1.15–2.15). In addition, rs2745709 in *miR-221/222* was significantly different (*P* < 0.001), and C/C-T/T genotype might be associated with a decreased risk of CIN in the overdominant model (OR = 0.66, 95% CI: 0.52–0.82).

**Table 3:** The inheritance model analysis of the ten SNPs in miRNA genes among Control and CIN groups

SNPs	Models	Genotypes	CIN n (%)	CON n (%)	OR (95% CI)	*P* value	AIC	BIC
rs1292037	Codominant	T/T	138 (31.7)	329 (33.0)	1.00	0.003	1753.7	1769.5
		T/C	237 (54.5)	465 (46.6)	0.82 (0.64–1.06)			
		C/C	60 (13.8)	204 (20.4)	1.43 (1.01–2.02)			
	Dominant	T/T	138 (31.7)	329 (33.0)	1.00	0.640	1763.1	1773.6
		T/C-C/C	297 (68.3)	669 (67.0)	0.94 (0.74–1.20)			
	Recessive	T/T-T/C	375 (86.2)	794 (79.6)	1.00	0.002	1754.0	1764.5
		C/C	60 (13.8)	204 (20.4)	1.61 (1.17–2.20)			
	Overdominant	T/T-C/C	198 (45.5)	533 (53.4)	1.00	0.006	1755.7	1766.3
		T/C	237 (54.5)	465 (46.6)	0.73 (0.58–0.91)			
	Log-additive	–	–	–	1.12 (0.95–1.31)	0.180	1761.5	1772.0
rs13137	Codominant	A/A	138 (31.7)	327 (32.8)	1.00	0.005	1754.7	1770.5
		A/T	236 (54.2)	467 (46.8)	0.84 (0.65–1.08)			
		T/T	61 (14.0)	204 (20.4)	1.41 (1.00–2.00)			
	Dominant	A/A	138 (31.7)	327 (32.8)	1.00	0.700	1763.1	1773.7
		A/T-T/T	297 (68.3)	671 (67.2)	0.95 (0.75–1.21)			
	Recessive	A/A-A/T	374 (86.0)	794 (79.6)	1.00	0.003	1754.7	1765.2
		T/T	61 (14.0)	204 (20.4)	1.58 (1.15–2.15)			
	Overdominant	A/A-T/T	199 (45.8)	531 (53.2)	1.00	0.009	1756.5	1767.1
		A/T	236 (54.2)	467 (46.8)	0.74 (0.59–0.93)			
	Log-additive	–	–	–	1.12 (0.95–1.31)	0.180	1761.5	1772.0
rs2745709	Codominant	C/C	129 (29.7)	381 (38.2)	1.00	0.001	1751.6	1767.5
		C/T	249 (57.2)	467 (46.8)	0.64 (0.49–0.82)			
		T/T	57 (13.1)	150 (15.0)	0.89 (0.62–1.28)			
	Dominant	C/C	129 (29.7)	381 (38.2)	1.00	0.002	1753.5	1764.1
		C/T-T/T	306 (70.3)	617 (61.8)	0.68 (0.54–0.87)			
	Recessive	C/C-C/T	378 (86.9)	848 (85.0)	1.00	0.340	1762.4	1772.9
		T/T	57 (13.1)	150 (15.0)	1.17 (0.84–1.63)			
	Overdominant	C/C-T/T	186 (42.8)	531 (53.2)	1.00	< 0.001	1750.0	1760.6
		C/T	249 (57.2)	467 (46.8)	0.66 (0.52–0.82)			
	Log-additive	–	–	–	0.87 (0.73–1.02)	0.090	1760.4	1770.9

The association of the genotypes of the 10 SNPs with CIN and CC was evaluated using inheritance model analysis (Table 4). In *miR-21*, our results revealed a significant difference in rs1292037 (*P* = 0.003) between these two groups, and the T/T-C/C genotype might be associated with an increased risk of CC in the overdominant model (OR = 1.43, 95% CI: 1.12–1.81). The rs13137 also showed a significant difference (*P* = 0.004), and the A/A-T/T genotype might be associated with an increased risk of CC in the overdominant model (OR = 1.42, 95% CI: 1.12–1.80). In *miR-221/222*, the rs2745709 exhibited a significant difference (*P* = 0.003), and the T/T-C/C genotype might be associated with an increased risk of CC in the overdominant model (OR = 1.44, 95% CI: 1.13–1.83). In *miR-126*, rs2297537 and rs2297538 showed significant differences in the log-additive and recessive models, respectively (*P* = 0.003 and 0.001), and 2C/C + C/G in rs2297537 and G/G-A/G in rs2297538 genotype might be associated with an increased risk of CC (OR = 0.71, 95% CI: 0.57–0.89 and OR = 2.83, 95% CI: 1.52–5.25). In contrast, no significant difference in the 10 SNPs between the CIN and control groups were detected (data not shown).

**Table 4:** The inheritance model analysis of the ten SNPs in miRNA genes among CIN and Cervical cancer groups

SNPs	Models	Genotypes	CC	CIN	OR (95 CI)	*P* value	AIC	BIC
rs1292037	Codominant	T/T	268 (36.1)	138 (31.7)	1.00	0.010	1548.3	1563.5
		T/C	339 (45.6)	237 (54.5)	1.36 (1.04–1.77)			
		C/C	136 (18.3)	60 (13.8)	0.86 (0.59–1.24)			
	Dominant	T/T	268 (36.1)	138 (31.7)	1.00	0.130	1553.3	1563.4
		T/C-C/C	475 (63.9)	297 (68.3)	1.21 (0.94–1.56)			
	Recessive	T/T-T/C	607 (81.7)	375 (86.2)	1.00	0.042	1551.5	1561.6
		C/C	136 (18.3)	60 (13.8)	0.71 (0.51–0.99)			
	Overdominant	T/T-C/C	404 (54.4)	198 (45.5)	1.00	0.003	1547.0	1557.1
		T/C	339 (45.6)	237 (54.5)	1.43 (1.12–1.81)			
	Log-additive	–	–	–	1.00 (0.84–1.18)	0.970	1555.6	1565.7
rs13137	Codominant	A/A	268 (36.1)	138 (31.7)	1.00	0.011	1548.5	1563.7
		A/T	338 (45.5)	236 (54.2)	1.36 (1.04–1.77)			
		T/T	137 (18.4)	61 (14)	0.86 (0.60–1.25)			
	Dominant	A/A	268 (36.1)	138 (31.7)	1.00	0.130	1553.3	1563.4
		A/T-T/T	475 (63.9)	297 (68.3)	1.21 (0.94–1.56)			
	Recessive	A/A-A/T	606 (81.6)	374 (86)	1.00	0.048	1551.7	1561.8
		T/T	137 (18.4)	61 (14)	0.72 (0.52–1.00)			
	Overdominant	A/A-T/T	405 (54.5)	199 (45.8)	1.00	0.004	1547.1	1557.3
		A/T	338 (45.5)	236 (54.2)	1.42 (1.12–1.80)			
	Log-additive	–	–	–	1.00 (0.84–1.18)	0.990	1555.6	1565.7
rs2745709	Codominant	C/C	260 (35)	129 (29.7)	1.00	0.010	1548.4	1563.6
		C/T	358 (48.2)	249 (57.2)	1.40 (1.07–1.83)			
		T/T	125 (16.8)	57 (13.1)	0.92 (0.63–1.34)			
	Dominant	C/C	260 (35)	129 (29.7)	1.00	0.059	1552.0	1562.2
		C/T-T/T	483 (65)	306 (70.3)	1.28 (0.99–1.65)			
	Recessive	C/C-C/T	618 (83.2)	378 (86.9)	1.00	0.085	1552.6	1562.8
		T/T	125 (16.8)	57 (13.1)	0.75 (0.53–1.05)			
	Overdominant	C/C-T/T	385 (51.8)	186 (42.8)	1.00	0.003	1546.5	1556.7
		C/T	358 (48.2)	249 (57.2)	1.44 (1.13–1.83)			
	Log-additive	–	–	–	1.04 (0.87–1.24)	0.690	1555.4	1565.6
rs2297537	Codominant	C/C	459 (61.8)	302 (69.4)	1.00	0.009	1548.1	1563.3
		C/G	254 (34.2)	125 (28.7)	0.75 (0.58–0.97)			
		G/G	30 (4)	8 (1.8)	0.41 (0.18–0.90)			
	Dominant	C/C	459 (61.8)	302 (69.4)	1.00	0.008	1548.5	1558.6
		C/G-G/G	284 (38.2)	133 (30.6)	0.71 (0.55–0.92)			
	Recessive	C/C-C/G	713 (96)	427 (98.2)	1.00	0.032	1551.0	1561.1
		G/G	30 (4)	8 (1.8)	0.45 (0.20–0.98)			
	Overdominant	C/C-G/G	489 (65.8)	310 (71.3)	1.00	0.052	1551.8	1562.0
		C/G	254 (34.2)	125 (28.7)	0.78 (0.60–1.00)			
	Log-additive	–	–	–	0.71 (0.57–0.89)	0.003	1546.6	1556.7
rs2297538	Codominant	G/G	535 (72)	315 (72.4)	1.00	0.002	1544.6	1559.9
		A/G	191 (25.7)	93 (21.4)	0.83 (0.62–1.10)			
		A/A	17 (2.3)	27 (6.2)	2.70 (1.45–5.03)			
	Dominant	G/G	535 (72)	315 (72.4)	1.00	0.880	1555.6	1565.7
		A/G-A/A	208 (28)	120 (27.6)	0.98 (0.75–1.28)			
	Recessive	G/G-A/G	726 (97.7)	408 (93.8)	1.00	0.001	1544.4	1554.5
		A/A	17 (2.3)	27 (6.2)	2.83 (1.52–5.25)			
	Overdominant	G/G-A/A	552 (74.3)	342 (78.6)	1.00	0.092	1552.7	1562.9
		A/G	191 (25.7)	93 (21.4)	0.79 (0.59–1.04)			
	Log-additive	–	–	–	1.13 (0.91–1.40)	0.280	1554.4	1564.6

## Discussion

Many studies have reported that SNPs in miRNAs are related to various diseases, especially cancers [22, 23, 28, 29]. In the current study, we investigated the association of 10 SNPs in the *miR-21*, *miR-26b*, *miR-221/222* and *miR-126* genes with CIN and CC in Han Chinese women. According to our results, rs2297538 and rs2297537 in *miR-126*, rs1292037 in *miR-21*, and rs2745709 in *miR-221/222* are associated with CIN or CC susceptibility in the Han Chinese population.

miR-21 acts as an oncogene in cancer by regulating signalling pathways involved in cancer development [30]. In 2015, Xu et al. reported that overexpression of miR-21 inhibited expression of the target gene PTEN in CC cell lines, and promoted the proliferation, migration and invasion of CC cells [31]. In the current study, we found a significant difference in rs1292037 and rs13137 in *miR-21* between CIN and CC groups in the overdominant model (*P* = 0.003 and 0.004). Moreover, we observed a significant difference in rs1292037 and rs13137 in *miR-21* between CIN and control groups in the recessive model (*P* = 0.002 and 0.003). In 2018, Zhang et al. investigated correlations of *miR-21* gene rs1292037 and rs13137 with chemosensitivity to cisplatin plus paclitaxel and prognosis before CC surgery [25], the results showed that rs1292037 is associated with chemoresistance to cisplatin plus paclitaxel as well as CC prognosis [25]. In 2017, Du et al. detected the sensitivity of CC cells to paclitaxel and found that inhibiting expression of miR-21 could suppress cell proliferation and colony formation via PTEN/AKT pathway regulation, therefore improving the PTX sensitivity of CC cells [32]. Thus, rs1292037 might play an important role in the association of miR-21 with the development of CIN or CC, in addition to the chemosensitivity of CC. Moreover, in 2015, Chacon-Cortes et al. performed an association study to assess correlation between rs1292037 and rs13137 in the *miR-21* gene and breast cancer, unfortunately, no association was detected [33]. The reason for the discrepancy between the study of Chacon-Cortes et al. and the current study might due to different types of cancers examined. These two SNPs should be investigated in other cancer types, such as lung cancer. Consequently, the function of these two SNPs in the development of CC should be investigated in future studies.

Several studies have found that miR-126 is usually under expressed in human colorectal cancer [34], breast cancer [35] and CC [18]. Additionally, our previous study found that rs4636297 in miR-126 was associated with CIN and CC in a Han Chinese population [23]. In the current study, we found that the distribution of the *miR-126* rs2297538 genotypes and rs2297537 alleles were significantly different between CIN and CC groups, which indicated that these two SNPs might play important roles in the progression of CIN to CC. The rs2297537 and rs2297538 are located in the promoter region, and are only 194 base pairs away. The former might serve as a binding site for transcription factors, as predicted by the NIH database [36]. Although the function of rs2297537 and rs2297538 is still unknown, our results indicated that they might be associated with CIN progression to CC through influencing transcription factors binding efficiency at the initiation of miR-126 transcription.

In 2013, Gocze et al. reported that miR-221 is overexpressed in squamous cell carcinoma, regardless of HPV status and clinical grade [16]. Similarly, miR-222 was reported to be up-regulated in CC tissues [13]. In the current study, we found a significant difference of rs2745709 in the *miR-221/222* between the CIN and control groups (*P* = 0.001). Few studies have reported the association between rs2745709 and CC, with a lack of reports about the relationship between rs2745709 and expression of *miR-221*. Thus, the role of rs2745709 in CC remains unclear, and its function in CC should be investigated.

In the current study, we investigated the association of SNPs in *miR-21*, *miR-26b, miR-221/222*, and *miR-126* among healthy controls, CIN patients and CC patients in a Han Chinese population. Our data showed that rs2297538 in *miR-126*, rs1292037 in *miR-21* and rs2745709 in *miR-221/222* are associated with the development of CIN and CC. In the future, larger-scale and functional SNP studies are required to better clarify and examine the role of these SNPs in the susceptibility, resistance and development of CC.

## Data Availability

The data generated during the current study are available to any scientist wishing to use them for non-commercial purpose from the corresponding author on reasonable request. However, the clinical data might be available without the privacy data of participates in the current study.
